# Surgical treatment of progressive cauda equina compression caused by spontaneous spinal subdural hematoma: A case report

**DOI:** 10.1097/MD.0000000000014598

**Published:** 2019-03-22

**Authors:** Xigong Li, Ge Yang, Zhiqiang Wen, Xianfeng Lou, Xiangjin Lin

**Affiliations:** a Department of Orthopedic Surgery, The First Affiliated Hospital, School of Medicine, Zhejiang University, Hangzhou; b Department of Orthopedics, Hunan Children's Hospital, The Pediatric Academy of University of South China, Hunan, China.

**Keywords:** cauda equine syndrome, hematoma, magnetic resonance imaging, spontaneous, subdural

## Abstract

**Rationale:**

Spontaneous spinal subdural hematoma (SSDH) without an underlying pathology is a very rare condition. The treatment protocol for SSDH is early diagnosis and treatment before irreversible damage to neural tissue. However, there is no agreement on the etiopathogenesis, as well as the need for surgery to treat spontaneous SSDH. Here, we report a rare case of spontaneous SSDH with progressive deterioration and symptoms of cauda equina syndrome after ineffective conservative treatment.

**Patient's concern:**

A 38-year-old male patient presented with sudden lower back and bilateral leg pain.

**Diagnosis:**

A magnetic resonance imaging (MRI) scan on the third day after the onset of symptoms revealed a subdural hematoma from L1 to S1, presenting as hyperintensities on T1 weighted sequences and hypointensities to isointensities on T2 weighted sequences.

**Intervention:**

Laminectomy and subdural evacuation were performed immediately.

**Outcomes:**

An abnormal ligamentum flavum was observed intraoperatively. A histological examination revealed extravasation of blood in the degenerated ligamentum flavum. Postoperatively, the lower limb pain improved immediately. At the 6-month follow-up, the pain and numbness of the lower limb disappeared, and the muscle strength of both legs recovered completely with normal gait.

**Lessons:**

Spontaneous SSDH with ligamentum flavum hematoma was caused by a sudden increase of intravenous pressure, resulting from a marked surge in the intra-abdominal or intrathoracic pressure. Consecutive MRI scans provided valuable information, leading to a diagnosis of spontaneous SSDH. The treatment protocol for spontaneous SSDH should be determined based on the location and stage of the hematoma, as well as the subject's neurological status.

## Abstract 2

Supplemental Digital Content is available in the text

## 1 Introduction

Spontaneous spinal subdural hematoma (SSDH) is an uncommon disease. Several predisposing factors associated with the development of SSDH, including trauma,^[1,2]^ coagulation abnormalities,^[3–5]^ lumbar puncture^[6,7]^ and vascular malformations.^[8]^ However, SSDH can also occur spontaneously in the absence of these underlying conditions. Currently, only a few cases of spontaneous SSDH have been reported, and its etiopathogenesis remains unclear.

The clinical presentation of SSDH is diverse, and it involves back and radicular pain and neurological deficits arising from progressive nerve root or spinal cord compression. The treatment protocol for SSDH is early diagnosis and treatment before irreversible damage to neural tissue. However, there is no agreement on the need for surgery to treat spontaneous SSDH. Several investigators have proposed prompt surgical evacuation, regardless of the preoperative neurological status.^[3,9–12]^ By contrast, others have suggested conservative treatment, even in high-risk cases with neurological deficits, contending that SSDH can resolve spontaneously.^[13–15]^

Here, we report a rare case of spontaneous SSDH with progressive cauda equina compression. The patient presented with deteriorating neurological deficits after ineffective conservative treatment and underwent laminectomy and hematoma evacuation. We also discuss the etiopathogenesis, clinical features and treatment protocol for spontaneous SSDH. This report was approved by the Ethics Committee of The First Affiliated Hospital (approval number: 00978199), College of Medicine, Zhejiang University (Hangzhou, China).

## 2 Case report

**Figure 1:**
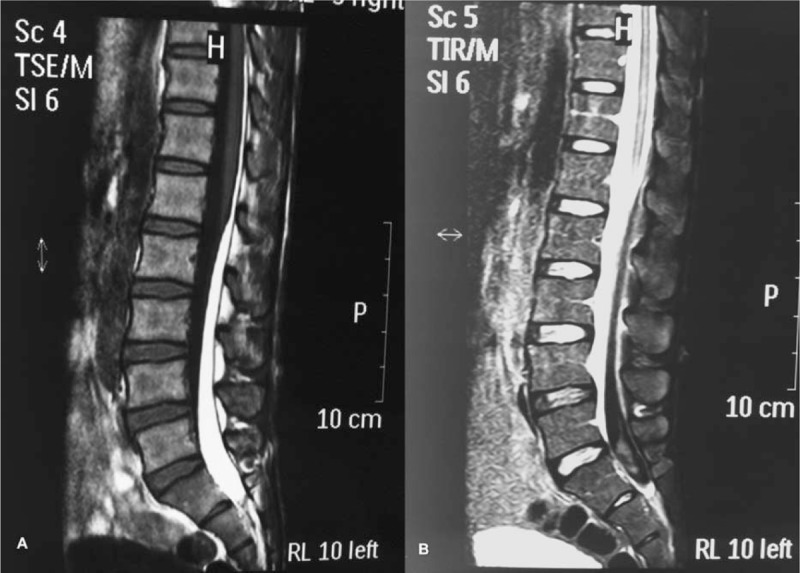
MRI scan of the lumbar spine at local hospital. Sagittal T1-weighted (A) and T2-weighted (B) images revealed a large subdural hematoma, extending from L1 to S1, presents as hyperintense signal on T1 weighted sequences and hypointense to isointense signal on T2 weighted sequences. MRI = magnetic resonance imaging.

A 38-year-old man experienced sudden onset of lower back pain when he bent down to pick up a newspaper off the floor. There was no history of trauma, cardiovascular disease, and bleeding disorders as well as no experience with drugs. The lower back pain worsened, and bilateral leg pain developed within 3 days. The subject received a medical examination and a magnetic resonance imaging (MRI) scan of the lumbar spine at a local hospital. MRI images revealed a large subdural hematoma, extending from L1 to S1 in the sagittal view and presenting as hyperintensities on T1 weighted sequences and hypointensities to isointensities on T2 weighted sequences (Fig. 1). A conservative treatment plan was decided upon at a local hospital because there were no severe neurological deficits. However, the symptoms worsened progressively with lower extremity muscle weakness, gait disturbance, and numbness in the saddle area 15 days after onset. Therefore, the subject was transferred to our center for further examination and management.

**Figure 2:**
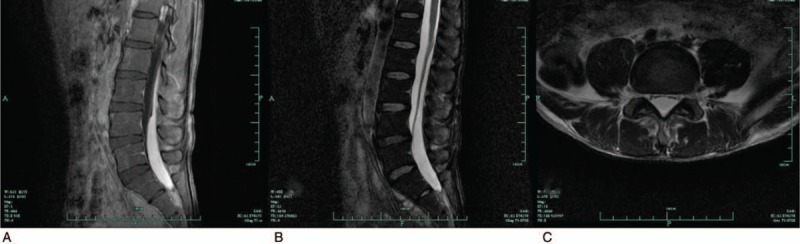
Repeated MRI scan of the lumbar spine at our center 15 days after onset. The size of subdural hematoma increased from L4 to S1 levels in the sagittal views, presenting as hyperintense signals on both T1-weighted and fat-suppressed T2-weighted sequences (A, B), and the cauda equina was dorsally compressed in the axial views (C). MRI = magnetic resonance imaging.

A neurological examination demonstrated paresthesia and pain below the L4 dermatome and motor weakness at grade 4 on the right lower limb and grade 3 on the left lower limb. The bilateral Achilles’ tendon reflex decreased, and the straight-leg-raising test was positive for both lower limbs. There was no evidence of bowel and bladder disturbances, and no pathological reflexes were identified. Laboratory tests revealed an acceptable platelet count and normal coagulation. A repeated MRI scan of the lumbar spine revealed an increase in the size of the subdural hematoma from L4 to S1 in the sagittal views, and the cauda equina was dorsally compressed in the axial views (Fig. 2). Furthermore, there was a change in the signal intensity of the subdural hematoma, which presented as hyperintensities on both T1-weighted and fat-suppressed T2-weighted sequences (Fig. 2).

**Figure 3:**
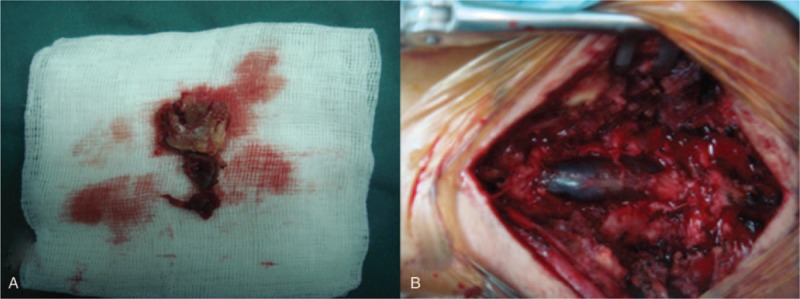
Intra-operative findings showed dark discoloration of the resected ligamentum flavum (A), and the dura was observed (B).

**Figure 4:**
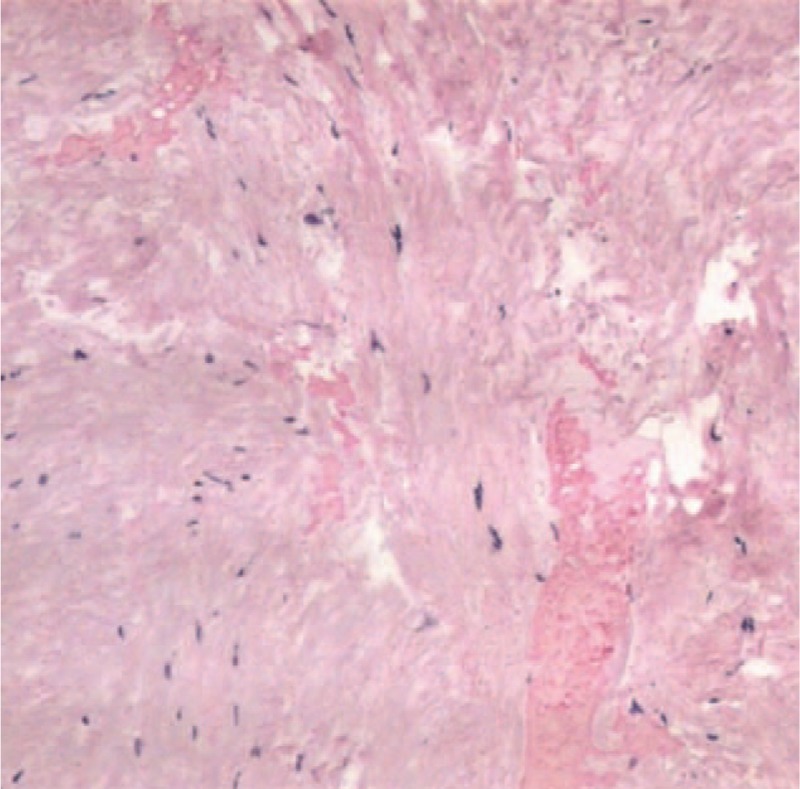
Histological findings showed erythrocytes and hemosiderin deposition around the degenerative fibrous tissues.

Subdural evacuation of the hematoma was performed immediately to improve the neurological symptoms. After bilateral L5-S1 laminectomy, the ligamentum flavum sustained hypertrophy and turned brown, and it was resected intraoperatively (Fig. 3). A pathological examination showed degeneration and formation of a new hematoma within the ligament (Fig. 4). The dura mater was tough and discolored (Fig. 3). After opening the dura with a longitudinal midline incision, dark brown blood drained spontaneously (Video 1). The region was irrigated with saline until the cerebrospinal fluid was clear and the nerve roots were visible through the intact arachnoid membrane. There was no evidence of a vascular abnormality and no bleeding from the subarachnoid space. After closing the dura with running lock sutures, its pulsatile motion was restored (Video 2).

**Figure 5:**
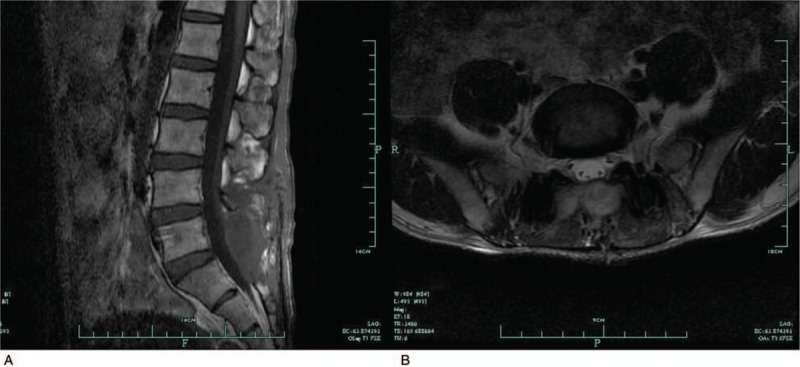
Post-operative MRI images. Sagittal T1-weighted (A) and axial T2-weighted (B) images showed the uncompressed cauda equina. MRI = magnetic resonance imaging.

Postoperatively, the lower limb pain improved immediately. An MRI revealed complete drainage of the chronic SDH (Fig. 5). The patient was discharged 1 week after surgery. At the 6-month follow-up, the pain and numbness of the lower limb pain disappeared, and the muscle strength of both legs recovered completely with normal gait.

## 3 Discussion

Spontaneous SSDH without an underlying pathology is a very rare condition. Presently, there is no agreement on the source of bleeding and the regulatory mechanism of spontaneous SSDH. Although the subdural space is though to be a cavity between the inner layer of the dura and the underlying arachnoid, an electron microscopic study showed no evidence of a cavity at the dura-arachnoid junction.^[16]^ By contrast, the inner layer of the dura was directly apposed to the outer layer of the arachnoid. Compared with the external dura and arachnoid, we observed flattened fibroblasts, few cell junctions and little extracellular matrix collagen at the inner border of the cell layer. During stress, the unique structural features of the dura-arachnoid junction promote the creation of a space, and the extra-arachnoidal vessels crossing the inner layer of the dura can then rupture simultaneously, resulting in the formation of a subdural hematoma.

One regulatory mechanism of spontaneous SSDH explains that a sudden surge in the intravenous pressure can increase the intra-abdominal or intrathoracic pressure.^[11,17–20]^ The Valsalva maneuver, which is frequently used during physical exercises, such as in squatting and diving, rapidly increases the intrathoracic and intra-abdominal pressures and can result in the formation of a subdural hematoma.^[18,20]^ Yamada et al reported a case of subdural hematoma in a postpartum female and concluded that an increased intra-abdominal pressure during pregnancy caused the formation of the subdural hematoma.^[19]^ Ozdemir et al also reported a case of spontaneous SSDH concomitant with a bilateral incarcerated inguinal hernia.^[11]^ They inferred that bowel obstruction increased the intra-abdominal pressure, resulting in increased intravascular pressure and ruptured vessels, which caused a spontaneous spinal hemorrhage.

Our intraoperative findings showed an abnormal ligamentum flavum, and a histological examination confirmed a hemorrhage within the degenerated ligamentum flavum. The normal ligamentum flavum consists of poorly vascularized, dense, elastic and collageneous fibers with more capillaries in the median portion than in the marginal and central portions of the ligamentum. However, the capillary number increases dramatically during degeneration, thereby increasing the prevalence of hemorrhage within the ligamentum flavum.^[21]^ Other investigators proposed that spontaneous vessel rupture caused by elevated intravenous and intra-abdominal pressures results in hemorrhage, and possibly hematoma within the ligamentum flavum.^[22–25]^ We concluded that an increased pressure of the vessels within the dura-arachnoid and ligamentum flavum caused by an acute increase of the intra-abdominal pressure resulted in SSDH concomitant with a ligamentum flavum hematoma in our subject.

The clinical symptoms of SSDH in the lumbar spine often mimic those of an intervertebral disc herniation with pain in the lower back and lower extremity. MRI is the method of choice for differentiating SSDH from herniated discs and other diseases. It also can better identify the location and the extent of the hemotoma as well as the extent of the compression of the spinal cord or cauda equina. The clinical symptoms of SSDH are similar to those previously reported for intraspinal hematoma.^[26,27]^ Classically, acute SSDH (1–3 days) is isointense to minimally hypointense on T1-weighted images and markedly hypointense on T2-weighted images, because of the presence of deoxyhemoglobin. Early subacute SSDH (3–7 days) is hyperintense on T1-weighted images and hypointense on T2-weighted images, because of the conversion of intracellular deoxyhemoglobin to methemoglobin. However, late subacute SSDH (1–2 weeks) shows high signal intensities on both T1-weighted and T2-weighted images due to the presence of methemoglobin. For our subject with subacute SSDH, the MRI signals provided important information that guided our treatment protocol.

Early diagnosis and prompt surgical decompression lead to good clinical outcomes. However, there is no agreement on the need for surgery to treat spontaneous SSDH.^[12,17]^ Only a few cases of spontaneous recovery have been reported after conservative treatment.^[13–15]^ Conservative management is recommended in cases with the progressive improvement of symptoms or the presence of mild neurological deficits. However, our subject presented with deteriorating cauda equine symptoms and underwent surgical decompression due to ineffective conservative treatment. Therefore, the subject's neurological status was the major deciding factor for surgical decompression. To predict clinical outcomes, Komiyama defined 2 types of SSDH. In cases of ventral SSDH, neurologic improvement is often evident, because the cerebrospinal fluid dilutes the ventral hematoma to achieve spontaneous decompression.^[28]^ In cases of dorsal SSDH (i.e., the subject of this study), surgical decompression is often necessary. These findings indicate that the location of the spinal hematoma dictates if surgery is required. Other investigators have suggested that SSDH confined to the lumbar spine can be successfully treated by percutaneous drainage.^[29,30]^ However, this procedure markedly increased the risk of iatrogenic injury to blood vessels, and the removal of the spinal hematoma with percutaneous drainage was extremely difficult in our subject. In conclusion, we recommend that clinicians treating subjects with spontaneous SSDH consider the location and stage of the hematoma as well as the neurological status of the subject.

## Author contributions

**Data curation:** Xigong Li, Ge Yang.

**Formal analysis:** Ge Yang.

**Investigation:** Xigong Li.

**Supervision:** Xigong Li.

**Writing – original draft:** Xigong Li, Ge Yang.

**Writing – review & editing:** Xigong Li, Zhiqiang Wen, Xianfeng Lou, Xiangjin Lin.

## Supplementary Material

Supplemental Digital Content

## Supplementary Material

Supplemental Digital Content
